# Comparison of Troponin in a Prehospital Blood Sample and Risk Scores

**DOI:** 10.3390/diagnostics16172688

**Published:** 2026-08-23

**Authors:** Juan F. Delgado Benito, Ana Ramos-Rodríguez, Enrique Castro-Portillo, Carlos del Pozo Vegas, Raúl López-Izquierdo, Francisco T. Martínez Fernández, Santiago Otero de la Torre, Miguel Á. Castro Villamor, Ancor Sanz-García, Francisco Martín-Rodríguez

**Affiliations:** 1 Prehospital Critical Care, Emergency Medical Services, Gerencia Regional de Salud de Castilla y León, 47001 Valladolid, Spain; jdelgado@saludcastillayleon.es (J.F.D.B.); fmartinezf@saludcastillayleon.es (F.T.M.F.); sotero@saludcastillayleon.es (S.O.d.l.T.); francisco.martin.rodriguez@uva.es (F.M.-R.); 2 Emergency Department, Hospital Universitario Río Hortega, Gerencia Regional de Salud de Castilla y León, 47001 Valladolid, Spain; aramosrodri@saludcastillayleon.es (A.R.-R.); rlopeziz@saludcastillayleon.es (R.L.-I.); 3 Primary Health Care Unit, Gerencia Regional de Salud de Castilla y León, 47001 Valladolid, Spain; ecastrop@saludcastillayleon.es; 4 Emergency Department, Hospital Clínico Universitario de Valladolid, Gerencia Regional de Salud de Castilla y León, 47007 Valladolid, Spain; cpozove@saludcastillayleon.es; 5 Faculty of Medicine, Universidad de Valladolid, 47005 Valladolid, Spain; 6 Valladolid Health Research Institute (IBioVALL), 47005 Valladolid, Spain; 7 Technological Innovation Applied to Health Research Group (ITAS Group), Faculty of Health Sciences, University of Castilla-La Mancha, 45600 Talavera de la Reina, Spain; ancor.sanz@uclm.es; 8 Group of Healthcare Assessment, Instituto de Investigación Sanitaria de Castilla-La Mancha (IDISCAM), 45004 Toledo, Spain

**Keywords:** prehospital emergency care, cardiac troponin I, acute coronary syndrome, risk stratification, major adverse cardiac events, emergency medical services, point-of-care testing

## Abstract

**Background/Objectives:** Prehospital identification of non-ST-elevation acute coronary syndrome remains unresolved, and available risk scores incorporate troponin categorically. We compared the discriminative performance of cardiac troponin I measured in a blood sample obtained at first prehospital medical contact, analysed as a continuous variable, against the HEART, GRACE, and TIMI scores for predicting 30-day major adverse cardiac events. **Methods:** We conducted a prospective cohort study in 333 adults with non-traumatic chest pain suggestive of non-ST-elevation acute coronary syndrome, which was attended by two physician-staffed advanced life support units. Venous blood was drawn at first medical contact and analysed later in the laboratory; the results were not available during patient care. All three scores used this same determination as their troponin component. The primary outcome was a composite of all-cause mortality, type 1 myocardial infarction, and unplanned coronary revascularisation at 30 days. **Results:** The primary outcome occurred in 82 patients (24.6%). Prehospital troponin discriminated better (area under the curve 0.916, 95% CI 0.875–0.952) than TIMI (0.817), GRACE (0.767), and HEART (0.721); all comparisons were *p* < 0.001. An exploratory rule-out threshold of 80 ng/L identified 61 patients (18.3%) with no events (sensitivity and negative predictive value 100%, exact 95% CI 95.6–100 and 94.1–100), whereas the ≥780 ng/L stratum showed an event rate of 69.4%. **Conclusions:** A blood sample obtained at first prehospital medical contact carries substantial prognostic information that clinical scores discard by categorising it. Because it was analysed subsequently in the laboratory, these findings do not evaluate a real-time strategy; they support development and validation of point-of-care devices.

## 1. Introduction

Prehospital electrocardiographic identification and direct catheterisation laboratory activation have transformed the care of ST-elevation myocardial infarction. Prehospital identification of non-ST-elevation acute coronary syndrome, by contrast, remains an unresolved challenge: more than 30% of these patients present with a normal or non-diagnostic electrocardiogram, so the twelve-lead tracing alone is insufficient for risk stratification [1].

Consequently, patients with suspected non-ST-elevation acute coronary syndrome are routinely transported to the emergency department irrespective of their actual risk, contributing to crowding and delaying care for those who require urgent intervention. European and North American guidelines recommend coronary angiography within 24 h in high-risk patients [1,2], and every minute of delay to reperfusion is associated with higher mortality [3,4]. Early identification, before hospital arrival, therefore, remains an unmet need. Dedicated chest pain units and accelerated diagnostic pathways have improved the structured evaluation of these patients once they reach hospital [5], but no equivalent pathway exists before arrival, where the decision on destination must nonetheless be taken.

Obtaining a venous blood sample at first medical contact is feasible in physician-staffed emergency medical systems and shortens emergency department length of stay when integrated into the care pathway [6,7]. Cardiac troponin measured in the prehospital setting is associated with short-term outcomes [8,9], and recent studies have evaluated rule-out strategies based on a single measurement combined with clinical scores [10,11,12].

Point-of-care devices are evolving rapidly [13], but their deployment in the field requires first establishing what prognostic information a sample obtained at first medical contact carries, irrespective of where and when it is analysed.

The HEART [14,15], GRACE [16], and TIMI [17] scores were derived in hospital cohorts with access to serial sampling and a complete diagnostic workup, which limits their transportability to a setting where decisions must be made within a single encounter. All three incorporate troponin categorically, a practice that discards quantitative information and degrades predictive performance [18,19].

To our knowledge, no prospective study has simultaneously compared, within the same patient sample and from the same analytical measurement, a single prehospital troponin determination treated as a continuous variable against all three scores.

The primary objective was to compare the discriminative performance of cardiac troponin I measured in a sample obtained at first prehospital medical contact, analysed as a continuous variable, against the HEART, GRACE, and TIMI scores for the prediction of 30-day major adverse cardiac events.

Secondary objectives were to derive decision thresholds specific to the analytical platform used, to evaluate performance against type 1 acute myocardial infarction alone, and to characterise agreement between the prehospital determination and hospital high-sensitivity assays.

## 2. Materials and Methods

### 2.1. Study Design and Setting

This was a prospective cohort study: consecutive eligible patients were identified and enrolled at first medical contact, before any outcome had occurred, and all were followed forward for a predefined period of 30 days with complete outcome ascertainment and no losses to follow-up. The study was conducted through collaboration between two advanced life support (ALS) units and two university tertiary hospitals in Valladolid (Castilla y León, Spain), serving a reference population of 433,221 inhabitants, between June 2024 and December 2025. Each ALS unit is staffed by a physician, a registered nurse, and two emergency medical technicians. The participating hospitals were Hospital Clínico Universitario de Valladolid and Hospital Universitario Río Hortega.

One hospital has a catheterisation laboratory available 24 h a day; from the second centre, patients requiring urgent coronary intervention undergo emergent direct transfer to that laboratory. Both centres, therefore, provide effective access to coronary intervention.

Cases were enrolled consecutively and continuously (24/7/365) within the parent study “Clinical characterization of acute pain in prehospital critical care: novel biomarkers and therapeutic targets”. The study was conducted in accordance with the STARD 2015 [20] and STROBE [21] reporting guidelines (completed checklists are provided in Tables S2 and S3) and was approved by the institutional review boards of the participating healthcare system (reference 2022/07/1127). Written informed consent was obtained from all participants.

### 2.2. Participants

Eligible patients were adults (≥18 years) with non-traumatic chest pain suggestive of non-ST-elevation acute coronary syndrome, assessed on scene by the ALS physician and transported to the emergency department.

Exclusion criteria were age below 18 years, traumatic chest pain, pregnancy, terminal illness with an advance directive, out-of-hospital cardiac arrest, discharge at scene, inability to obtain a venous blood sample, and absence of informed consent. No a priori sample size calculation was performed; the analysis included all consecutive eligible patients attended during the study period.

### 2.3. Prehospital Data Collection

All participating providers completed mandatory training in the study protocol, with formal competency verification before enrolment. The ALS nurse recorded epidemiological variables and vital signs using a LIFEPAK^®^ 15 monitor-defibrillator (Stryker/Physio-Control Inc., Redmond, WA, USA) at first medical contact, before any therapeutic intervention.

The time of emergency call, first medical contact, departure from scene, and hospital arrival was recorded for every patient. The time of blood sampling coincided with the time of first medical contact in all 333 cases. Time from symptom onset was estimated by the attending physician and recorded in minutes; estimates clustered at a limited number of values (thirteen distinct values across the cohort), reflecting the rounding inherent to clinical estimation. This coarse resolution should be borne in mind when interpreting the time-stratified analyses. A twelve-lead electrocardiogram was obtained at first medical contact and interpreted by the attending physician; echocardiography was not performed in the prehospital setting.

### 2.4. Prehospital Cardiac Troponin I Measurement

Venous blood was drawn at first medical contact into sodium citrate tubes, one of the anticoagulants specified by the assay manufacturer. Tubes were refrigerated on board at 6 °C. A designated investigator from each unit transported the samples daily, including weekends, under continuous refrigeration to the laboratory of the Faculty of Medicine, Universidad de Valladolid, where they were analysed as whole blood within 24 h of collection, in accordance with the manufacturer’s specifications. Samples were never frozen and were discarded after analysis.

Cardiac troponin I was measured with the AFIAS Tn-I Plus (Boditech Med Inc., Gangwon-do, Republic of Korea), an automated fluorescence immunoassay, from 100 µL of sample with a result available after 12 min. The manufacturer reports the following analytical characteristics: limit of blank 4 ng/L, limit of detection 10 ng/L, limit of quantification 30 ng/L, measuring range 10–15,000 ng/L, and a 99th percentile upper reference limit of 40 ng/L derived from 125 apparently healthy volunteers, which coincides with the lowest concentration showing a coefficient of variation ≤10%. An independent evaluation has reported comparable performance [22]. The assay does not meet IFCC criteria for high-sensitivity designation, and its limit of quantification exceeds the 99th percentile upper reference limits of contemporary high-sensitivity assays (17–27 ng/L) [13], so it does not resolve concentrations within the range in which those assays operate.

Because analysis was performed off-site and after the index episode, the results were not generated during patient care. Prehospital providers, receiving hospital teams and the cardiologists who established the discharge diagnosis were, therefore, structurally unable to access them, independent of any blinding procedure.

### 2.5. Hospital Laboratory Troponin

The first in-hospital determination on arrival was performed at Hospital Clínico Universitario de Valladolid (Abbott Architect STAT High Sensitive Troponin-I) and Hospital Universitario Río Hortega (Siemens Atellica IM TnIH), both high-sensitivity cardiac troponin I platforms, applying manufacturer-specified sex-specific 99th percentile upper reference limits. Cardiac troponin T was not measured; both platforms quantify cardiac troponin I.

### 2.6. Clinical Risk Scores

The HEART [14,15], GRACE [16], and TIMI [17] scores were calculated retrospectively by the study data manager from prospectively recorded variables. Full components and scoring of each instrument are provided in Table S1. The prehospital troponin concentration was used as the troponin component of all three scores, ensuring that the four strategies compared are based on the same analytical measurement and that observed differences reflect the handling of troponin—continuous versus categorised—rather than different biomarkers [18,19].

In the primary analysis, the HEART troponin component was derived from the manufacturer’s 99th percentile upper reference limit (40 ng/L), in accordance with the original definition of the score. Because this limit proved non-discriminatory in the present cohort, a pre-specified sensitivity analysis applied the threshold that maximised HEART performance within the cohort, a scenario deliberately favourable to the comparator.

### 2.7. Outcomes

The primary outcome was the occurrence of major adverse cardiac events within 30 days of the index prehospital encounter, defined as a composite of all-cause mortality, type 1 acute myocardial infarction, and unplanned coronary revascularisation (percutaneous coronary intervention or coronary artery bypass grafting).

Type 1 myocardial infarction was diagnosed by the attending cardiologist during the index admission, in accordance with the Fourth Universal Definition of Myocardial Infarction [23], and recorded in the electronic health record. Investigators extracted and coded these diagnoses at 30-day follow-up together with coronary angiography and revascularisation data.

A pre-specified sensitivity analysis replaced all-cause mortality with cardiovascular death according to standardised endpoint definitions [24], and a secondary analysis used type 1 myocardial infarction alone as the outcome.

### 2.8. Statistical Analysis

Continuous variables are reported as median (interquartile range) and compared with the Mann–Whitney U test; categorical variables as frequency (percentage) and compared with the chi-square or Fisher exact test as appropriate.

Discrimination was assessed by the area under the receiver operating characteristic curve, with 95% confidence intervals obtained by bootstrapping (2000 resamples). Correlated curves were compared with the DeLong test. Decision thresholds were derived within the cohort—rule-out at 100% sensitivity, Youden optimum, and rule-in at ≥95% specificity—with bootstrap optimism correction. Clinical utility was assessed by decision curve analysis [25]. Confidence intervals for sensitivity, specificity, and predictive values were obtained by bootstrapping, except where the point estimate was 100%, for which exact binomial (Clopper–Pearson) intervals were used. Because these thresholds were both derived and evaluated within the same cohort, without external validation, they are regarded as exploratory.

Analyses were performed in Python 3.12 (NumPy, pandas, SciPy, scikit-learn) with a fixed random seed (20260729). The analysis code is available from the corresponding author on reasonable request.

Generative artificial intelligence (Claude Opus 5, Anthropic; extended thinking mode) was used to assist in developing the statistical analysis code, in auditing the study dataset, and in coding free-text discharge diagnoses into predefined outcome categories. Of the 333 records, 176 were screened for potential type 1 myocardial infarction on the basis of a coronary discharge diagnosis, elevated hospital troponin, coronary intervention, or death within 30 days; the remaining 157 had hospital troponin concentrations below the 99th percentile of both laboratory platforms and were classified as non-cases without further review. Of the 176 screened, 135 were assigned with high or intermediate confidence by predefined text criteria, and 41 with ambiguous free-text descriptions were adjudicated case by case by the corresponding author, blinded to the prehospital troponin result. All diagnoses originated from the attending cardiologist during the index admission; no diagnosis was assigned by the tool. Duplicate independent adjudication was not performed. All analytical output was reviewed by the investigators, and the principal results were independently reproduced by a co-author from the study dataset. The authors take full responsibility for the content of this manuscript.

## 3. Results

### 3.1. Study Population

Of 576 patients assessed on scene, 333 met the eligibility criteria and were enrolled (Figure S1). All had a prehospital blood sample obtained at first medical contact and complete 30-day follow-up. The primary composite outcome occurred in 82 patients (24.6%): 53 type 1 myocardial infarctions (15.9%), 31 unplanned coronary revascularisations (9.3%), and 12 deaths within 30 days (3.6%), of which eight were adjudicated as cardiovascular.

Baseline characteristics of both groups are shown in Table 1.

### 3.2. Discrimination for 30-Day Major Adverse Cardiac Events

Prehospital cardiac troponin I concentrations were markedly higher in patients who experienced the primary outcome (median 2885 ng/L, IQR 1098–7510) than in those who did not (110 ng/L, IQR 80–320; *p* < 0.001). All three clinical scores also differed significantly between groups: HEART 8 (7–9) versus 7 (6–7), GRACE 145 (123–175) versus 110 (85–135), and TIMI 4 (3–5) versus 2 (1–3); all *p* < 0.001.

**Figure 1:**
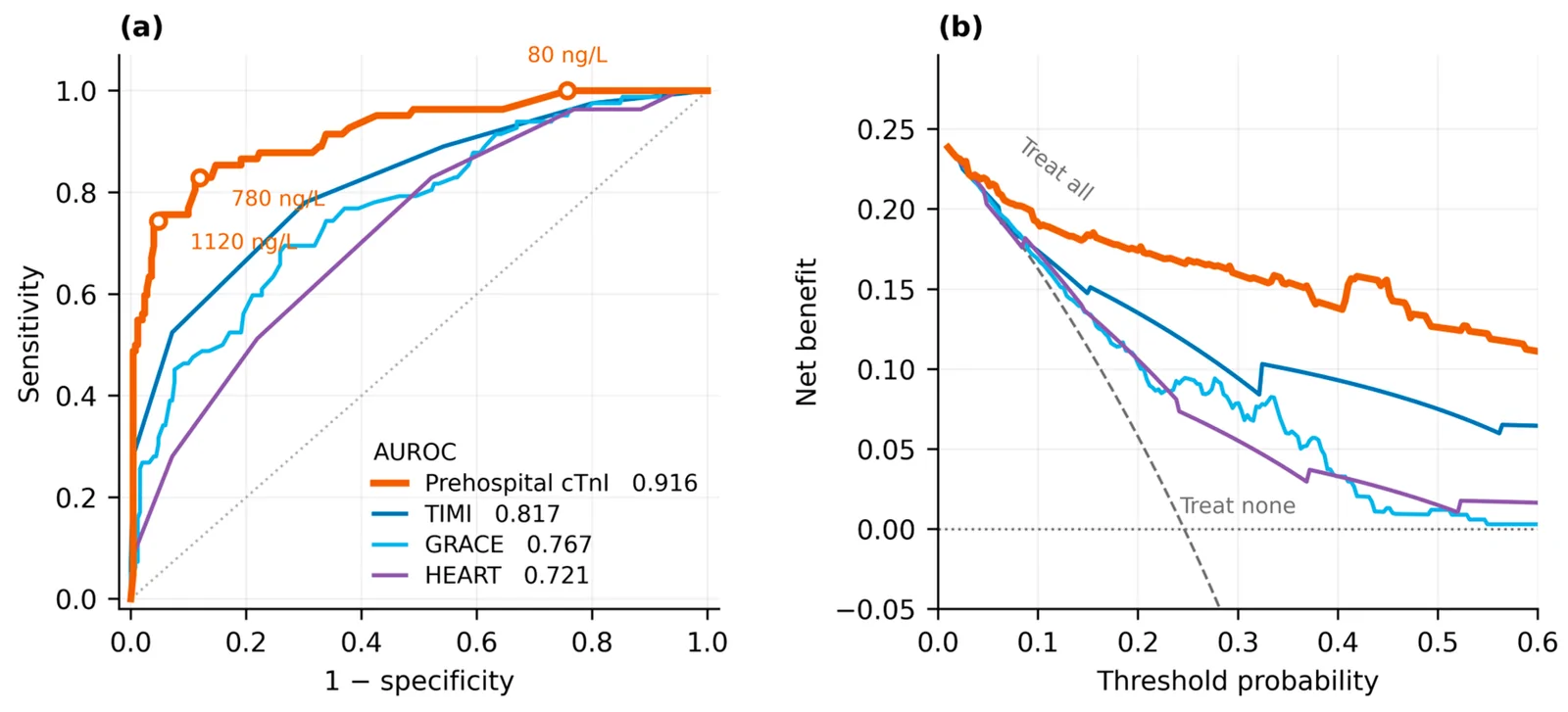
Discrimination and clinical utility. (**a**) Receiver operating characteristic curves for prehospital cardiac troponin I and the HEART, GRACE, and TIMI scores; open circles mark the cohort-derived rule-out (80 ng/L), Youden (780 ng/L), and rule-in (1120 ng/L) thresholds. (**b**) Decision curve analysis showing net benefit across threshold probabilities, with predicted probabilities obtained by logistic calibration of each predictor.

Prehospital troponin, analysed as a continuous variable, discriminated the primary outcome better than any of the three scores (Table 2, Figure 1a). Decision curve analysis showed greater net benefit for troponin across the clinically relevant range of threshold probabilities (Figure 1b).

### 3.3. Risk Stratification and Derived Thresholds

Because the assay’s limit of quantification lies above the upper reference limits of contemporary high-sensitivity assays, decision thresholds were derived within the cohort rather than adopted from published values (Table 3). A rule-out threshold of 80 ng/L identified 61 patients (18.3%) with no events at 30 days, yielding sensitivity and negative predictive value of 100% (exact 95% CI 95.6–100% and 94.1–100%, respectively) and specificity of 24.3% (19.2–29.6). A rule-in threshold of 1120 ng/L achieved specificity of 95.2% (92.5–97.6) and positive predictive value of 83.6% (74.6–91.9). Both thresholds were derived and evaluated within the same cohort and should be regarded as exploratory pending external validation.

**Figure 2:**
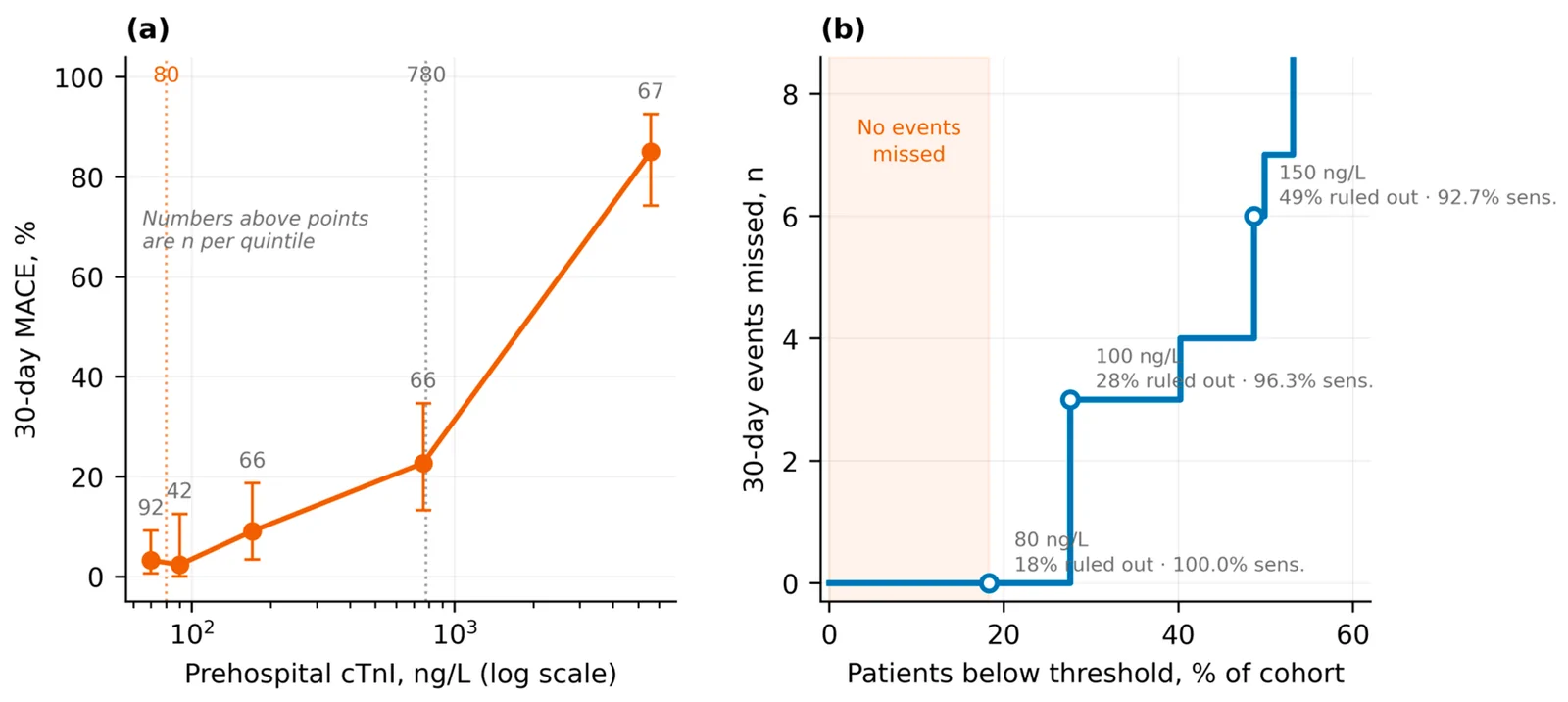
Prehospital troponin concentration and 30-day outcome. (**a**) Event rate by quintile of troponin concentration, with 95% confidence intervals; numbers above points indicate patients per quintile, and dotted lines mark the rule-out (80 ng/L) and Youden (780 ng/L) thresholds. (**b**) Trade-off between rule-out yield and safety across the full range of possible thresholds: the proportion of the cohort falling below each threshold is plotted against the number of 30-day events missed. The shaded area indicates the range within which no events are missed.

Event rates increased monotonically across quintiles of troponin concentration (Figure 2a). Applying the rule-out and Youden thresholds produced three strata with event rates of 0.0% (n = 61), 8.0% (n = 174), and 69.4% (n = 98). Across the full range of possible cut-offs, no threshold above 80 ng/L excluded further patients without missing events: raising the threshold to 100 ng/L would have ruled out 28% of the cohort at the cost of three missed events, and to 150 ng/L, 49% at the cost of six (Figure 2b).

### 3.4. Prehospital Time Intervals

Median time from symptom onset to blood sampling was 35 min (IQR 20–50), with no difference between patients with and without the primary outcome (*p* = 0.544). Median time from sampling to hospital arrival was 43 min (IQR 35–51), likewise similar between groups (*p* = 0.176). Median total prehospital time was 53 min (IQR 45–64).

Discrimination remained high across all time strata. By time from symptom onset, the AUROC was 0.958 (0.925–0.985) up to 30 min, 0.891 (0.812–0.955) between 31 and 60 min, and 0.768 (0.517–1.000) beyond 60 min. By time to hospital arrival, it was 0.965 (0.894–1.000), 0.951 (0.907–0.986), and 0.874 (0.800–0.934) for intervals of up to 30, 31 to 45, and more than 45 min, respectively. The 80 ng/L threshold missed no events in any time window.

An interaction term between time from symptom onset and troponin concentration was statistically significant (*p* = 0.004) but lost significance when the 25 patients with onset beyond 60 min were excluded (*p* = 0.054). Given the small size of that stratum and the categorical nature of the onset time record, this finding is not considered conclusive.

### 3.5. Sensitivity Analyses

The results were unchanged when cardiovascular death replaced all-cause death in the composite outcome (78 events; troponin AUROC 0.918). For type 1 myocardial infarction alone, troponin achieved an AUROC of 0.902, compared with 0.848 for TIMI, 0.775 for HEART, and 0.752 for GRACE.

Applying the manufacturer’s 99th percentile upper reference limit (40 ng/L) to the HEART troponin component assigned the maximum score to 199 patients (59.8%) and the minimum to 2, rendering the component non-discriminatory. When the component was instead assigned the threshold that maximised HEART performance within this cohort, the score reached an AUROC of 0.829; prehospital troponin remained superior (*p* < 0.001). Without its troponin component, the HEART score achieved an AUROC of 0.638.

The interval between sample collection and analysis was not associated with the ratio of prehospital to hospital troponin concentration (Spearman ρ = 0.037, *p* = 0.50). Agreement between the prehospital assay and hospital high-sensitivity assays was moderate (ρ = 0.478, *p* < 0.001); 245 patients (73.6%) had hospital troponin concentrations below the prehospital assay’s limit of quantification. The first in-hospital troponin achieved an AUROC of 0.821 for the primary outcome.

## 4. Discussion

### 4.1. Main Findings

This study shows that, in a prospective cohort of 333 patients attended for suspected non-ST-elevation acute coronary syndrome by a physician-staffed emergency medical service, a single cardiac troponin I determination obtained at first medical contact and analysed as a continuous variable discriminated 30-day major adverse cardiac events better than the HEART, GRACE, and TIMI scores, with an area under the curve of 0.916 versus 0.721–0.817. Differences were statistically significant against all three scores, and decision curve analysis showed greater net benefit across the clinically relevant range of threshold probabilities [25].

One feature of the design merits emphasis: all three scores were calculated using the same analytical measurement as their troponin component. The observed difference, therefore, does not reflect different biomarkers but the handling of the same information—continuous versus categorised into three tiers. This constitutes an empirical demonstration of the predictive cost of dichotomising continuous predictors [18,19].

### 4.2. Relation to Previous Evidence

Previous prehospital studies have evaluated point-of-care troponin combined with clinical scores, predominantly aiming to safely rule out low-risk patients [10,26,27,28]. A recent individual patient data meta-analysis confirms the utility of a single measurement in this setting [12].

Our work offers a complementary approach: rather than testing a rule-out strategy, it quantifies how much prognostic information the sample carries and compares it simultaneously with the three most widely used scores within the same patient series. To our knowledge, no previous comparison has done so from a single analytical measurement shared by all predictors.

The performance observed is consistent with the reported association between prehospital troponin and short-term outcomes [8,9], and with results obtained in paramedic-staffed systems [11,29].

### 4.3. Score Transportability and Assay Characteristics

The secondary finding merits particular attention. When the manufacturer’s 99th percentile upper reference limit (40 ng/L) was applied to the HEART troponin component, 59.8% of patients received the maximum score and only two the minimum: the component ceased to discriminate, and the score fell to an area under the curve of 0.721. When the component was instead assigned the threshold that maximised the score’s own performance, it reached 0.829—and continuous troponin nonetheless remained superior.

The explanation is analytical. The limit of quantification of the assay used (30 ng/L) lies above the 99th percentile upper reference limits of contemporary high-sensitivity assays (17–27 ng/L) [13,30], so it does not resolve concentrations within the range in which those assays make their decisions. In our cohort, 73.6% of patients had hospital troponin concentrations below that limit.

The implication extends beyond this study: the transportability of a clinical score to the prehospital setting depends not only on the score itself but on the analytical characteristics of the available device. A component validated in the hospital environment may become uninformative when transferred to a platform with a different analytical floor. Thresholds derived here are platform-specific and are not transferable without cross-platform validation.

Risk stratification in non-ST-elevation acute coronary syndrome may also be influenced by factors beyond myocardial injury itself. In the present cohort, prehospital glucose concentrations were substantially higher in patients who experienced the primary outcome (median 139.5 versus 113.0 mg/dL, *p* < 0.001), an observation consistent with recent evidence that the stress hyperglycaemia ratio is associated with type 4a myocardial infarction and adverse outcomes in patients undergoing percutaneous coronary intervention [31]. We did not measure glycated haemoglobin and could not, therefore, compute this ratio, but the association we observed supports the broader argument that integrated models combining biomarker, clinical, and metabolic information may outperform any single measurement.

### 4.4. Clinical Implications

The results were not available during patient care, and this study, therefore, does not evaluate a real-time decision tool. The design tested here is a prehospital blood sample analysed subsequently in the laboratory, not point-of-care measurement in the field. The study demonstrates that the sample obtained at first medical contact carries substantial prognostic information; it does not demonstrate that access to this result in the ambulance would improve triage, catheterisation laboratory activation, emergency department bypass, or patient outcomes. Establishing that would require a prospective interventional trial. These findings support the development and validation of devices capable of generating such a result in the field [13,32].

The stratification obtained illustrates this potential: 18.3% of patients fell below the rule-out threshold with no events at 30 days, whereas the highest stratum concentrated an event rate of 69.4%. In this cohort, the median interval between blood sampling and hospital arrival was 43 min, which quantifies the window during which such information would become available ahead of hospital assessment. In a network such as the one studied—one centre with a permanently available catheterisation laboratory and emergent direct transfer from the second—this interval might in principle allow the pathway to be activated in advance, although whether doing so would improve outcomes remains untested [3,4,33]. The reduction in emergency department length of stay reported when prehospital sampling is integrated into the care pathway points in the same direction [6,34].

One case in this series illustrates the conceptual limit of any single-biomarker strategy: a patient with type A aortic dissection and a fatal outcome had a prehospital concentration in the low range. Troponin cannot exclude non-coronary life-threatening causes of chest pain, and no rule-out strategy should be applied independently of clinical judgement.

### 4.5. Limitations

This study has several limitations.

First, the primary outcome includes type 1 myocardial infarction, the diagnosis of which rests on hospital troponin, a measurement correlated with the prehospital determination. This constitutes incorporation bias: a troponin-based predictor is used to predict an outcome that depends in part on troponin, which may inflate apparent discrimination. Two considerations bound its magnitude without eliminating it. The bias affects all four compared predictors, since the three scores incorporate the same determination as their troponin component, although not necessarily to the same degree: the continuous measurement retains more troponin information than the tiered components and may, therefore, be inflated to a greater extent, so that the magnitude of the observed difference could be overestimated. Second, hospital troponin, which does contribute to the diagnosis, performed less well (AUROC 0.821) than the prehospital measurement (0.916), which would be difficult to explain if the result were merely circular. Absolute estimates of discrimination should nonetheless be interpreted with caution.

Second, analysis was deferred, performed in the laboratory after the index episode. Although this provides structural blinding—no clinician could access the result, independent of any procedure—it precludes extrapolation to real-time clinical use. This study does not evaluate a prehospital decision strategy.

Third, decision thresholds were derived and evaluated within the same cohort, without external validation or internal split-sample validation, and are, therefore, exploratory and probably optimistic. No a priori sample size calculation was performed; all consecutive eligible patients were included. With 82 events, the precision of extreme estimates is limited: in particular, the 100% sensitivity and negative predictive value at the rule-out threshold carry exact lower confidence bounds of 95.6% and 94.1%, respectively, so a residual risk of missed events cannot be excluded.

Fourth, the assay does not meet IFCC criteria for high-sensitivity designation, and its limit of quantification exceeds the 99th percentile upper reference limits of contemporary high-sensitivity platforms. Agreement with hospital troponin was moderate (ρ = 0.478) [30]. The thresholds reported are specific to this platform and are not transferable to other troponin assays or to future point-of-care devices without cross-platform validation.

Fifth, a single determination was performed, without serial sampling, so the rise or fall pattern required by the universal definition of myocardial infarction could not be verified [23]. Diagnoses of type 1 myocardial infarction were established by the attending cardiologist during the index admission and coded by the investigators; 41 records with ambiguous free-text descriptions were adjudicated case by case by the corresponding author, blinded to the prehospital troponin result. Duplicate independent adjudication was not performed. The classification of these 41 cases, therefore, rests on the judgement of a single reviewer, and residual misclassification of the outcome cannot be excluded; this should be borne in mind when interpreting the outcome-specific analyses.

Sixth, time from symptom onset was estimated by the attending physician rather than measured, and the resulting values were coarsely resolved, which limits interpretation of the time-stratified analyses. Behavioural, psychosocial, and environmental factors—alcohol intake, sleep quality, mood disorders, or domestic exposures—were not recorded and may act as unmeasured confounders.

Seventh, the study was conducted in two units within a single region, in a physician-staffed model. This staffing is precisely what makes on-scene venous sampling possible, but it limits generalisability to systems staffed exclusively by technicians, where obtaining a blood sample is not part of routine practice.

## 5. Conclusions

A single cardiac troponin I determination obtained at first prehospital medical contact and analysed as a continuous variable discriminates 30-day major adverse cardiac events better than the HEART, GRACE, and TIMI scores calculated from the same measurement. The prehospital sample, therefore, carries substantial prognostic information that these scores discard by categorising it. Because the sample was analysed subsequently in the laboratory and the result was not available during patient care, this study does not evaluate a real-time prehospital decision strategy, and the thresholds reported are exploratory and internally derived.

The performance of the troponin component of these scores depends critically on the analytical characteristics of the platform used, which conditions their transportability to the prehospital setting.

These findings support the development and prospective validation of devices capable of providing troponin quantification at the point of care, together with the derivation and external validation of platform-specific decision thresholds. Whether availability of such a result during prehospital care would improve triage, catheterisation laboratory activation or patient outcomes remains to be tested in an interventional study.

## Figures and Tables

**Table 1:** Baseline characteristics according to 30-day major adverse cardiac events (MACEs).

Characteristic	No MACE (n = 251)	MACE (n = 82)	*p* Value
Age, years	70.0 (57.0–81.0)	74.5 (64.0–83.0)	0.005
Female sex, n (%)	110 (43.8)	26 (31.7)	0.071
Systolic blood pressure, mmHg	140.0 (121.0–160.0)	138.5 (120.2–153.8)	0.505
Diastolic blood pressure, mmHg	81.0 (69.0–90.0)	83.5 (68.2–93.8)	0.490
Heart rate, beats/min	75.0 (64.0–89.0)	82.5 (66.0–96.8)	0.027
Respiratory rate, breaths/min	17.0 (14.0–20.0)	18.0 (16.0–20.8)	0.139
Oxygen saturation, %	97.0 (96.0–98.0)	96.0 (94.0–98.0)	0.007
Temperature, °C	36.0 (35.8–36.4)	36.0 (35.9–36.3)	0.904
Glucose, mg/dL	113.0 (97.5–138.5)	139.5 (112.2–176.8)	<0.001
Lactate, mmol/L	1.8 (1.2–2.4)	2.3 (1.9–3.3)	<0.001
Creatinine, mg/dL	0.9 (0.8–1.1)	1.1 (0.8–1.4)	0.020
Diabetes mellitus, n (%)	84 (33.5)	40 (48.8)	0.018
Hypertension, n (%)	161 (64.1)	66 (80.5)	0.009
Dyslipidaemia, n (%)	156 (62.2)	58 (70.7)	0.202
Current smoking, n (%)	55 (21.9)	24 (29.3)	0.226
Obesity, n (%)	73 (29.1)	31 (37.8)	0.180
Previous myocardial infarction, n (%)	102 (40.6)	54 (65.9)	<0.001

Values are median (interquartile range) unless otherwise stated. Comparisons by Mann–Whitney U test or chi-square test as appropriate.

**Table 2:** Discrimination of prehospital cardiac troponin I and clinical risk scores for 30-day major adverse cardiac events.

Predictor	AUROC	95% CI	*p* Value ^1^
Prehospital cTnI	0.916	0.875–0.952	Reference
TIMI score	0.817	0.762–0.870	0.0009
GRACE score	0.767	0.704–0.824	<0.001
HEART score	0.721	0.662–0.780	<0.001

^1^ DeLong test for paired areas under the curve, each score versus prehospital cardiac troponin I. AUROC, area under the receiver operating characteristic curve; CI, confidence interval; cTnI, cardiac troponin I.

**Table 3:** Diagnostic performance of prehospital cardiac troponin I at cohort-derived thresholds.

Strategy	Threshold (ng/L)	Sensitivity (95% CI)	Specificity (95% CI)	PPV (95% CI)	NPV (95% CI)
Rule-out	80	100 (95.6–100) ^1^	24.3 (19.2–29.6)	30.1 (24.9–36.0)	100 (94.1–100) ^1^
Youden optimum	780	82.9 (74.0–90.7)	88.0 (83.9–91.9)	69.4 (60.0–78.4)	94.0 (90.7–96.9)
Rule-in	1120	74.4 (64.4–83.5)	95.2 (92.5–97.6)	83.6 (74.6–91.9)	91.9 (88.4–95.1)

PPV, positive predictive value; NPV, negative predictive value. Values are percentages. Confidence intervals obtained by bootstrapping (2000 resamples). ^1^ Exact binomial (Clopper–Pearson) interval. Thresholds were derived and evaluated within the same cohort and are exploratory.

## Data Availability

The data presented in this study are available from the corresponding author upon reasonable request. The analysis code is available on the same basis.

## Supplementary Materials

The following supporting information can be downloaded at: https://www.mdpi.com/article/10.3390/diagnostics16172688/s1. Table S1: Components and scoring of the HEART, GRACE, and TIMI risk scores; Figure S1: Study flow diagram; Table S2: STARD 2015 checklist; Table S3: STROBE checklist.

## Abbreviations

The following abbreviations are used in this manuscript:

AUROC	Area under the receiver operating characteristic curve
CI	Confidence interval
cTnI	Cardiac troponin I
EMS	Emergency medical services
IQR	Interquartile range
MACE	Major adverse cardiac event
NPV	Negative predictive value
PPV	Positive predictive value
