# Supplementary material for: Comparison of Troponin in a Prehospital Blood Sample and Risk Scores
Source: Diagnostics (Basel). 2026 Aug 23;16(17):2688. doi: 10.3390/diagnostics16172688 (PMC13565609; doi:10.3390/diagnostics16172688)
Supplement: Supplementary file 1 [file diagnostics-16-02688-s001.zip › diagnostics-4500423-supplementary.pdf]

Supplementary Materials

Prehospital Troponin versus Risk Scores: A Prospective Cohort Study

Contents

Table S1. Components and scoring of the HEART, GRACE and TIMI risk scores

Figure S1. Study flow diagram

Table S2. STARD 2015 checklist

Table S3. STROBE checklist for cohort studies

Table S1. Components and scoring of the HEART, GRACE and TIMI risk scores.

HEART score

Component	0 points	1 point	2 points
History	Slightly suspicious	Moderately suspicious	Highly suspicious
Electrocardiogram	Normal	Non-specific repolarisation disturbance	Significant ST deviation
Age, years	< 45	45–64	≥ 65
Risk factors	No known risk factor	1–2 risk factors	≥ 3 risk factors or history of atherosclerotic disease
Troponin	≤ normal limit	1–3 × normal limit	> 3 × normal limit

Total score 0–10. Risk factors: diabetes mellitus, current or recent smoking, hypertension, dyslipidaemia, family history of coronary artery disease, obesity. In the present study the troponin component was derived from the manufacturer's 99th percentile upper reference limit (40 ng/L); a sensitivity analysis using an empirically optimised threshold is reported in the main text. References [13,14].

GRACE score

Component	Categories used	Points
Age, years	< 40 / 40–49 / 50–59 / 60–69 / 70–79 / ≥ 80	0 / 18 / 36 / 55 / 73 / 91
Heart rate, beats/min	< 70 / 70–89 / 90–109 / 110–149 / 150–199 / ≥ 200	0 / 7 / 13 / 23 / 36 / 46
Systolic blood pressure, mmHg	≥ 200 / 160–199 / 140–159 / 120–139 / 100–119 / 80–99 / < 80	0 / 11 / 26 / 37 / 47 / 58 / 63
Creatinine, mg/dL	0–0.39 / 0.40–0.79 / 0.80–1.19 / 1.20–1.59 / 1.60–1.99 / ≥ 2.00	5 / 8 / 11 / 14 / 23 / 31
Killip class	I / II / III / IV	0 / 21 / 43 / 64
Cardiac arrest at admission	No / Yes	0 / 43
ST-segment deviation	No / Yes	0 / 30
Elevated cardiac markers	No / Yes	0 / 15

Total score 1–372. Reference [15].

TIMI score

Component	Definition	Points
Age	≥ 65 years	1
Risk factors	≥ 3 risk factors for coronary artery disease	1
Known coronary artery disease	Prior stenosis ≥ 50%	1
Aspirin use	Within the preceding 7 days	1
Severe angina	≥ 2 episodes within the preceding 24 hours	1
ST deviation	≥ 0.5 mm on presenting electrocardiogram	1
Elevated cardiac markers	Above the upper reference limit	1

Total score 0–7. Reference [16].

Figure S1. Study flow diagram.

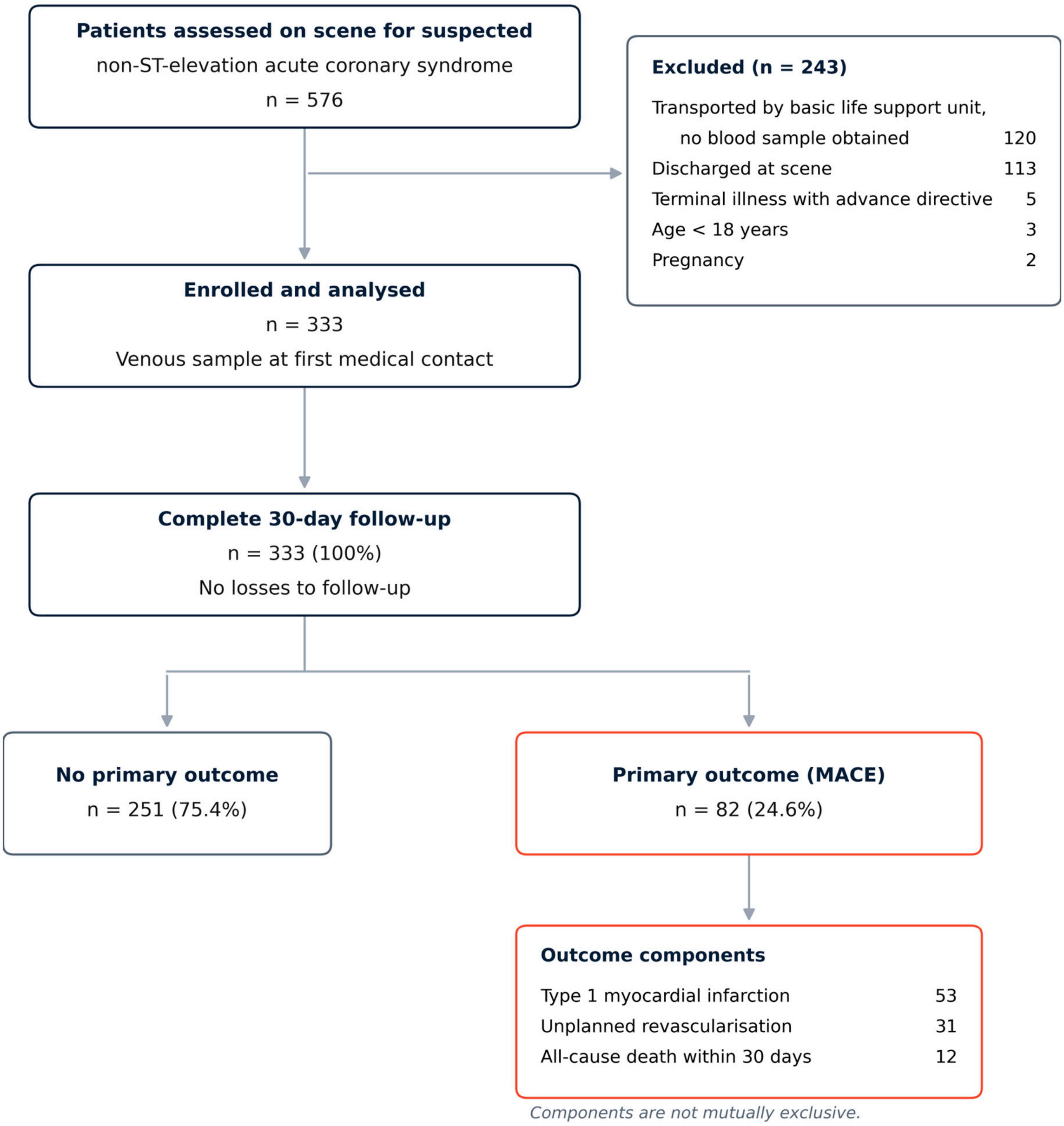

Flow of participants from initial on-scene assessment to outcome classification. Outcome components are not mutually exclusive: a patient could contribute more than one component to the composite outcome.

Table S2. STARD 2015 checklist for reporting studies of diagnostic accuracy.

Section	#	Item	Reported in section	Page
Title or abstract	1	Identification as a study of diagnostic accuracy using at least one measure of accuracy	Title; Abstract	
Abstract	2	Structured summary of design, methods, results and conclusions	Abstract	
Introduction	3	Scientific and clinical background	Section 1	
	4	Study objectives and hypotheses	Section 1	
Methods — study design	5	Whether data collection was planned before or after the index test and reference standard	Section 2.1 (prospective)	
Participants	6	Eligibility criteria	Section 2.2	
	7	On what basis potentially eligible participants were identified	Section 2.2	
	8	Where and when potentially eligible participants were identified	Section 2.1	
	9	Whether participants formed a consecutive, random or convenience series	Section 2.2 (consecutive)	
Test methods	10a	Index test, in sufficient detail to allow replication	Section 2.4	
	10b	Reference standard, in sufficient detail to allow replication	Sections 2.5, 2.7	
	11	Rationale for choosing the reference standard	Section 2.7	
	12a	Definition of and rationale for test positivity cut-offs of the index test	Sections 2.4, 2.8; Section 3.3 (thresholds exploratory, internally derived)	
	12b	Definition of and rationale for test positivity cut-offs of the reference standard	Section 2.7	
	13a	Whether clinical information and reference standard results were available to the performers of the index test	Section 2.4 (structurally blinded)	
	13b	Whether clinical information and index test results were available to the assessors of the reference standard	Section 2.4 (structurally blinded)	
Analysis	14	Methods for estimating or comparing measures of diagnostic accuracy	Section 2.8	
	15	How indeterminate index test or reference standard results were handled	Section 2.7	
	16	How missing data were handled	Section 3.1 (no missing data)	
	17	Any analyses of variability in diagnostic accuracy, distinguishing pre-specified from exploratory	Sections 2.8, 3.5	
	18	Intended sample size and how it was determined	Section 2.2 (no a priori calculation; consecutive sample)	
Results — participants	19	Flow of participants, using a diagram	Figure S1	
	20	Baseline demographic and clinical characteristics	Table 1	
	21a	Distribution of severity of disease in those with the target condition	Section 3.1; Figure S1	
	21b	Distribution of alternative diagnoses in those without the target condition	Section 3.1	
	22	Time interval and any clinical interventions between index test and reference standard	Sections 2.3, 3.4 (prehospital time intervals)	
Test results	23	Cross tabulation of the index test results by the results of the reference standard	Table 3	

	24	Estimates of diagnostic accuracy and their precision	Tables 2, 3 (with 95% confidence intervals); Figure 1
	25	Any adverse events from performing the index test or the reference standard	None occurred
Discussion	26	Study limitations	Section 4.5
	27	Implications for practice	Section 4.4
Other	28	Registration number and name of registry	Not registered
	29	Where the full study protocol can be accessed	Available on request
	30	Sources of funding and other support; role of funders	Funding statement

Page numbers refer to the submitted manuscript and should be completed once the final file is paginated.

Table S3. STROBE checklist for cohort studies.

Section	#	Recommendation	Reported in section	Page
Title and abstract	1	(a) Study design indicated in title or abstract; (b) informative and balanced summary	Title; Abstract	
Introduction	2	Scientific background and rationale	Section 1	
	3	Specific objectives, including any prespecified hypotheses	Section 1	
Methods	4	Key elements of study design presented early in the paper	Section 2.1	
	5	Setting, locations and relevant dates	Section 2.1	
	6	Eligibility criteria, sources and methods of participant selection	Section 2.2	
	7	Clearly defined outcomes, exposures and predictors	Sections 2.4–2.7	
	8	Sources of data and details of methods of assessment	Sections 2.3–2.5	
	9	Efforts to address potential sources of bias	Sections 2.4, 2.6; Section 4.5	
	10	How the study size was arrived at	Section 2.2 (no a priori calculation)	
	11	How quantitative variables were handled	Sections 2.6, 2.8	
	12	Statistical methods, including subgroup and sensitivity analyses	Section 2.8	
Results	13	Numbers of individuals at each stage; reasons for non-participation	Section 3.1; Figure S1	
	14	Characteristics of participants; missing data	Table 1; Section 3.1	
	15	Numbers of outcome events or summary measures	Section 3.1	
	16	Unadjusted estimates and their precision; category boundaries when continuous variables were categorised	Tables 2, 3 (with 95% confidence intervals)	
	17	Other analyses performed — subgroup and sensitivity analyses	Sections 3.4, 3.5	
Discussion	18	Key results summarised with reference to study objectives	Section 4.1	
	19	Limitations of the study	Section 4.5	
	20	Cautious overall interpretation of results	Sections 4.1–4.4	
	21	Generalisability of the study results	Section 4.5	
Other information	22	Source of funding and role of funders	Funding statement	

Page numbers refer to the submitted manuscript and should be completed once the final file is paginated.
